# Relevance theory for mapping cognitive biases in fact-checking: an argumentative approach

**DOI:** 10.3389/fpsyg.2024.1468879

**Published:** 2024-12-12

**Authors:** Mariavittoria Masotina, Elena Musi, Simeon Yates

**Affiliations:** ^1^Department of Communication and Media, University of Liverpool, Liverpool, United Kingdom; ^2^Digital Media and Society Institute, University of Liverpool, Liverpool, United Kingdom

**Keywords:** cognitive bias, relevance theory, argumentum model of topics, fact-checking, disinformation, misinformation

## Abstract

In the fast-paced, densely populated information landscape shaped by digitization, distinguishing information from misinformation is critical. Fact-checkers are effective in fighting fake news but face challenges such as cognitive overload and time pressure, which increase susceptibility to cognitive biases. Establishing standards to mitigate these biases can improve the quality of fact-checks, bolster audience trust, and protect against reputation attacks from disinformation actors. While previous research has focused on audience biases, we propose a novel approach grounded on relevance theory and the argumentum model of topics to identify (i) the biases intervening in the fact-checking process, (ii) their triggers, and (iii) at what level of reasoning they act. We showcase the predictive power of our approach through a multimethod case study involving a semi-automatic literature review, a fact-checking simulation with 12 news practitioners, and an online survey involving 40 journalists and fact-checkers. The study highlights the distinction between biases triggered by relevance by effort and effect, offering a taxonomy of cognitive biases and a method to map them within decision-making processes. These insights can inform trainings to enhance fact-checkers’ critical thinking skills, improving the quality and trustworthiness of fact-checking practices.

## 1 Introduction

It is well known that the spread of fake news across social media constitutes a major challenge for society, whether it is misinformation—inaccurate information spread without the intent to deceive—or disinformation—non-factual information spread with the intention to mislead (Wu et al., 2019). Fact-checking, the process of verifying the accuracy of news, has emerged in response to the rise of misinformation and disinformation. Third-party fact-checkers are professionals outside news media agencies who debunk false, misleading, or inaccurate claims and publish their findings (Graves and Amazeen, 2019). The number of fact-checking websites has grown from 11 in 2008 to 417 by June 2023, covering 100 countries (Stencel et al., 2023). This growth has led to efforts to establish standards and best practices, resulting in the 2015 launch of the International Fact-Checking Network (IFCN), which covers nearly one-third of active fact-checking organizations (*N* = 176).

Fact-checking has proven effective in correcting misinformation across different countries, with a lasting impact on audience beliefs (e.g., Walter et al., 2020; Porter and Wood, 2021; Carnahan and Bergan, 2022). The effectiveness of fact-checking depends on it being considered trustworthy (Amazeen, 2015), and some fact-checkers have been accused of being biased or partisan (Amazeen, 2013). This accusation must be addressed by IFCN signatories, as their standards require transparency about conflicts of interests to ensure trustworthiness. However, other factors than overt bias and partisanship could undermine fact-checkers’ trustworthiness. One such factor is cognitive bias. Cognitive biases have received less attention, even though the affordances of digital media potentially increase the likelihood of their influence when verifying information.

Cognitive biases are systematic patterns of thinking that lead to deviations from rational decision-making (Korteling and Toet, 2020). For example, cognitive biases have been found to affect individual’s choices about a medical treatment. Decisions varied depending on whether the same statistics are framed as probabilities of survival or of mortality (Veldwijk et al., 2016). People also overestimated the likelihood of an outcome based on the availability of similar cases in their memory (Lichtenstein et al., 1978; Pachur et al., 2012) or attributed positive characteristics to their ingroup and negative ones to their outgroup (Rabbie and Horwitz, 1969; Bauer and Hannover, 2020).

The rise of the Internet and social media has transformed the information landscape into an increasingly fast-paced, dense media content environment. Print newspaper circulation has declined sharply (UK Paliament, 2020; Statista, 2024), whereas online news and social media have gained popularity (European Parliament, 2023; Ofcom, 2023). News agencies can publish significantly more content online, allowing for continuous updates (PressGazette, 2023; Usearch, 2024). The border between news producers and consumers has blurred as online audiences actively contribute to news production, engage in debates about their accuracy, and use user-generated content as information sources (Jenkins, 2006; Ugille, 2017). Overall, the contemporary news landscape is characterized by a continuous stream of information from diverse and often contradictory sources. Participating in it requires rapid sense-making and communication. This increases the likelihood of cognitive overload and time pressure, making the intervention of cognitive biases more likely (e.g., Allred et al., 2016; Zong and Guo, 2022).

From its introduction (Tversky and Kahneman, 1973), the concept of cognitive biases has received significant attention. Over 200 biases have been identified as affecting decision-making in various contexts such as management, finance, medicine, law, and consumer behavior (Berthet, 2022; Manoogian and Benson, 2017; Monaco et al., 2024; Waldman, 2020). Nonetheless, little is known about the intervention of cognitive biases in the fact-checking process. Understanding their impact could inform the establishment of standards in this area, enhancing the perceived trustworthiness of fact-checkers and showing their commitment to impartiality at all levels. To accomplish this, it is essential to identify which biases are most likely to intervene, how these biases differ, and at what level of reasoning they operate.

In this paper, we propose and test a novel approach to categorize and map cognitive biases relevant to the fact-checking process. Our framework draws from three observations. First, fact-checkers must face challenges imposed by contextual factors (e.g., time constraints) and individual beliefs while pursuing impartiality in news verification. Second, relevance plays a pivotal role in guiding the fact-checking process. That is, the prioritization of news items for fact-checking will be determined by their relevance to the likely audience. Third, fact-checking is an argumentative process. By this, we mean that the evaluation of a news item as more or less accurate constitutes a standpoint that has to be supported by arguments.

In view of the first two observations, we adopt relevance theory (Sperber and Wilson, 1986; Wilson and Sperber, 2006) as a theoretical framework for understanding the mechanisms underlying cognitive biases in fact-checking. Accordingly, we classify cognitive biases based on the two components recognized as underpinning relevance in communication: cognitive effort, which we associate with contextual factors, and cognitive effect, which we link to individual beliefs. We hypothesize that:

*H1. In the context of the fact-checking process for the contemporary digital news media ecosystem, biases triggered by both “relevance by effort” and “relevance by effect” can influence the outcome of the process.*

Viewing fact-checking as an argumentative process allows us to reconstruct the argumentative scheme underlying fact-checkers’ decision-making. We propose to use the argumentum model of topics (Rigotti and Greco Morasso, 2010), a semantic and pragmatic theory to unpack the way we draw inferences through argumentative discourse, to map at what level of reasoning cognitive biases intervene. We hypothesize that:

*H2. The distinction between cognitive biases triggered by ‘relevance by effort’ and those triggered by ‘relevance by effect’ can be shown by reconstructing the inferential pathway of the decision-making process.*

As a first exploration of these two hypotheses, we report a case study involving news media practitioners. The results are based on a multi-method approach involving (1) a semi-automatic literature review, (2) a fact-checking simulation in which we observed behavior of fact-checkers, and (3) an online survey where we collected self-reported data by practitioners.

The rest of our contribution is organized as follows: Section 2 provides an overview of related studies about cognitive bias in the misinformation ecosystem; Section 3 presents our framework, by introducing fact-checking as a cognitive process (Section 3.1), previous biases classifications and relevance theory (Section 3.2), and the argumentum model of topics (Section 3.3). In Section 4, we describe our case study and discuss its results. Section 5 provides the conclusion of this paper.

## 2 Related studies

To our knowledge, the only contribution addressing cognitive biases in the fact-checking process is a recent review by Soprano et al. (2024). The authors considered a list of 221 biases from the scientific literature and identified those potentially relevant to truthfulness assessments by both non-expert assessors (e.g., crowd workers) and fact-checkers. To categorize these biases, they adapted a classification scheme based on information visualization research (Dimara et al., 2018) and they proposed 11 countermeasures grounded in existing research. The countermeasures primarily target researchers (e.g., advocating for randomized experimental designs) and non-expert assessors (e.g., emphasizing training to enhance accuracy and reduce bias). The authors suggest that further studies should observe real-case scenarios, as their study is a theoretical account and not focused on actual professional practice. Our contribution undertakes this task. We propose a novel approach to categorize and map cognitive biases relevant to professional fact-checking that we then empirically validate. From a theoretical perspective, we diverge from the classification of Dimara et al.’s (2018) as some tasks in information visualization are not applicable to fact-checking (e.g., “opinion reporting tasks”) and others are not considered (e.g., prioritizing information for fact-checking).

Within the broader information ecosystem, previous studies have focused on cognitive biases affecting the general audience, with results that are not directly applicable to professional fact-checking. Recent research has concentrated on three main aspects: (i) how biases affect fact-checking readers’ interpretations of content, (ii) how they influence the ways citizens independently seek evidence to verify news truthfulness, and (iii) how they shape initial impressions about the accuracy of news.

As to (i), Park et al. (2021) demonstrated that different fact-checking labels can influence citizens’ reactions, with claims labeled as ‘Lack of Evidence’ often perceived as false due to uncertainty aversion (Ellsberg, 1961). Additionally, confirmation bias (Wason, 1960) plays a role in interpreting labels like ‘Mostly True’ and contributes to the backfire effect of fact-checking on Facebook (Zollo, 2019). The findings are useful for improving fact-checkers’ dissemination practices but offer limited insights into the fact-checking process itself.

Concerning (ii), biases such as What You See Is All There Is bias (tendency to overlook alternative possibilities), anchoring bias (over-reliance on initial information), and overconfidence bias (overestimation of one’s abilities or knowledge) have been proven as obstacles to news consumers looking for additional evidence about news items (e.g., Piro and Anderson, 2018; French et al., 2023). This issue is not applicable to professional fact-checking where verification is, by default, the primary task. Nonetheless, Endsley (2018) suggests that disinformation exploits anchoring (Tversky and Kahneman, 1974) and confirmation bias. In this case, initial misinformation can bias subsequent information search and processing, reinforcing preexisting beliefs and expectations. This insight is potentially applicable also to the fact-checking process.

As to (iii), evidence demonstrates the impact of cognitive biases on the ability to distinguish between true and fake news due to the influence of initial impressions. Studies in psychology have consistently highlighted the impact of confirmation bias, the ‘overconfidence effect’, and type I biases. These lead to incorrect conclusions about relationships between unrelated phenomena (Jylhä et al., 2022; Saltor et al., 2023; Matute et al., 2015; Teovanović et al., 2021; Croce and Piazza, 2023; Lindeman et al., 2023; Martínez-Costa et al., 2023). Studies in social science research led to similar results. Confirmation bias and the ‘overconfidence effect’ have been found to have detrimental effects on recognizing fake news (e.g., Milovidov, 2018). Similar outcomes have been found for the ‘illusory truth effect’ where people tend to judge information as true if they encounter it repeatedly (Hasher et al., 1977; Croce and Piazza, 2023) and political bias where there is a tendency to favor certain political ideologies, perspectives, or parties over others (Tandoc et al., 2021; Sádaba et al., 2023).

A line of research about cognitive biases and disinformation has emerged also in the computer science field. It has given particular attention to biases facilitated by digital affordances and filter bubbles or echo-chambers phenomena where, in online environments, individuals are primarily exposed to contents that reinforce their existing views (Karduni et al., 2018; Morales-i-Gras, 2020; Ruffo et al., 2023). The studies confirmed the effect of multiple biases. These included confirmation bias and illusory truth effects, alongside within-group bias where people favor information that benefits one’s social group. Other biases included the bandwagon effect, which is the tendency to align with the opinion that is perceived as being shared by the majority (Mutz, 1998), and third person effect which is the tendency to believe that disinformation affects another group more than the one the individual belongs to (Davison, 1983). A further identified bias is the false consensus effect, which is the tendency for individuals to think their beliefs are widely accepted (Ross et al., 1977). All these biases can facilitate the sharing of online misinformation content.

All the research presented in this section enhances our understanding of how cognitive biases operate within the misinformation ecosystem. However, most findings are not directly applicable to the professional fact-checking process. This paper aims to bridge this gap by proposing and testing a framework tailored to the practices of professional fact-checkers.

## 3 Our framework

### 3.1 Context

In the current digital information ecosystem, news-making operates as an argumentative process (Musi et al., 2023). A given news item is a piece of information whose truth validity is negotiated among individuals. News claims (standpoints) are presented within narratives and supported by arguments to persuade the audience about their soundness. Audiences actively engage in the debate by providing feedback across various platforms, either supporting the standpoints produced with further arguments or challenging them with counterarguments or alternative viewpoints.

Fact-checking is part of the same debate, with professionals writing fact-checks and intervening in controversies regarding the accuracy of news and their associated arguments. They provide counterarguments against false or inaccurate information or address claims made against the accuracy of news when they are true. These counterarguments shall then be acknowledged by the audience and guide decisions. Both news-making and fact-checking are decision-making processes as they entail deciding what sources of information to trust in support of a world view. However, the structure of the decision-making is more constrained in fact-checking as the standpoint is ultimately an evaluation of the accuracy of news.

The decision-making process underlying the news evaluation can be reconstructed in the fact-checking report. While specific practices can vary across organizations, these reports typically synthetize the key content in the headline and expand on supporting arguments in subsequent paragraphs. For instance, on FullFact’s website (https://fullfact.org/)—one of the UK’s most known fact-checking agencies—we find the headline: ‘BBC Breakfast gets the increase in people waiting for NHS treatment wrong’. Here, the standpoint is that the news item is inaccurate. The reasoning behind this evaluation is *by definition* (Zarefsky, 1997; Minielli, 2010): misreporting statistics entails inaccurate news. As BBC Breakfast misinterpreted data, then the piece of news is inaccurate.

The expression of standpoints of fact-checkers and of their arguments is the result of multiple decisions addressing three main stages: news selection, evidence retrieval, and fact-checking report writing. These phases require a series of decisions to be made. What (potential fake) news shall be prioritized? What sources are necessary and/or sufficient to verify the claims? Where to look for evidence? What to highlight in a fact-checking report? The International Fact-Checking Network (IFCN) offers standards in this regard that include: importance and reach of news shall guide their selection; all key claims should be checked against diverse, non-partisan sources, with a preference for firsthand (e.g., data reports and witnesses); fact-checking reports should explain the rationale behind claim selection, cite relevant sources with associated links, and disclose any conflict of interest (International Fact-Checking Network, 2024). However, these guidelines allow for interpretation. For example, about what determines news importance or relevance of sources.

The reality of fact-checking practice is complex, and fact-checkers face numerous challenges while taking decisions. Cognitive biases are more likely to influence decision-making in situations of cognitive overload (e.g., Allred et al., 2016) or time pressure (e.g., Zong and Guo, 2022), and fact-checkers often operate within a rapid and packed news media environment. Fact-checkers often encounter a wide range of potential and conflicting sources and inaccurate claims, which increases cognitive load. On the other hand, providing timely and relevant disconfirmation is crucial to prevent the spread of misinformation and discourage audiences from integrating inaccurate information into their beliefs (Walter and Murphy, 2018; Walter and Tukachinsky, 2020). Additionally, each potential inaccuracy in fact-checking can cause a crisis of trust in the audience. Establishing clear procedures to mitigate cognitive biases can enhance audiences’ trust and prevent counterarguments from disinformation actors.

### 3.2 Relevance theory for cognitive biases classification

Fact-checking as a communicative process is a decision-making process centered upon relevance. When writing their report, fact-checkers must assess the relevance of the news they prioritize, of the evidence picked during the verification, and understand which arguments are the most relevant to support their standpoint on news accuracy. Although fact-checkers aim to be as systematic as possible, the determination of relevance can be influenced by individual beliefs (e.g., on what constitutes a relevant source) and contextual factors (e.g., lack of time) at each stage.

The importance of both individual and contextual factors makes it difficult to apply existing classifications of cognitive bias to fact-checking. Taxonomies like Oreg and Bayazit's (2009) classify a subset of biases based on individual differences and motivations, overlooking contextual factors. In contrast, taxonomy of Benson’s (2016) categorizes biases by the contextual factors that enable them (‘too much information,’ ‘need to act fast,’ ‘not enough meaning,’ ‘what should I remember?’), without considering the role of individual beliefs. In contrast, relevance theory accounts for this distinction. Relevance theory, developed by Sperber and Wilson in the 1980s (Sperber and Wilson, 1986; Sperber and Wilson, 1986) within pragmatics and cognitive science, views communication as a psychological and linguistic phenomenon at once. According to this theory, human cognition is geared toward maximizing relevance in communication. This framework accounts for bias as the result of mental mechanisms prioritizing attention to inputs deemed most relevant and processing them to enhance their relevance. In this framework, relevance amounts to the difference between the cognitive effort undertaken versus the cognitive effects achieved. Cognitive effort refers to the mental resources and processing capacity expended by individuals when interpreting and understanding incoming information or stimuli. Cognitive effect refers to the mental outcomes or changes in understanding that result from processing and interpreting that incoming information or stimuli. In the authors’ words, “other things being equal, the greater the cognitive effect achieved, and the smaller the mental effort required, the more relevant this input will be to you at the time” (Wilson and Sperber, 2006, p.5).

This framework allows us to draw parallels with the challenges faced by fact-checkers. Fact-checkers, to cope with the fact proliferation of disinformation, seek to reduce cognitive load (cognitive effort) while maximizing the update to their worldview (cognitive effect) selecting and processing the sources of information needed to assess the veridicality of news. Here, we propose relevance theory as an approach to classify bias encountered in fact-checking, and we hypothesize that:

*H1. Biases triggered by both “relevance by effort” and “relevance by effect” can influence the outcome of the fact-checking process.*

### 3.3 Argumentum model of topics for mapping cognitive biases

To decide which component of relevance (effort or effect) is at stake at what stage of fact-checking, it is paramount to focus on news verification in practice, rather than from a normative point of view. For instance, according to the IFCN, the reach and importance of claims should guide the choice of the news to fact-check. What it is advocated is, therefore, relevance by effect: the more popular and impactful a (fake) claim is, the more likely it will (misleadingly) influence people’s world views. However, it would be naive to neglect the impact that time and multimodality have on the choice of the news to prioritize, pushing for relevance by effort. In other words, these sets of motivations are in competition and need to be observed in context. The same applies to the phases of source selection and report writing. However, the reasoning in place is opaque as the public has access to the fact-checking product rather than the process. To focus on the process, it is necessary to work hand in hand with fact-checkers asking them to think aloud their choices.

Reconstructing the arguments used by practitioners when asked to justify these decisions can indicate if a bias is interfering with the decision-making process and at what level of the inferences made to draw a standpoint (e.g., “I will pick this news”/“I trust this news”; “this is a true news”). Furthermore, mapping the inferential processes leading to a choice allows us to explore whether biases activated by cognitive effort versus cognitive effect act upon different levels of the reasoning process.

**Figure 1:**
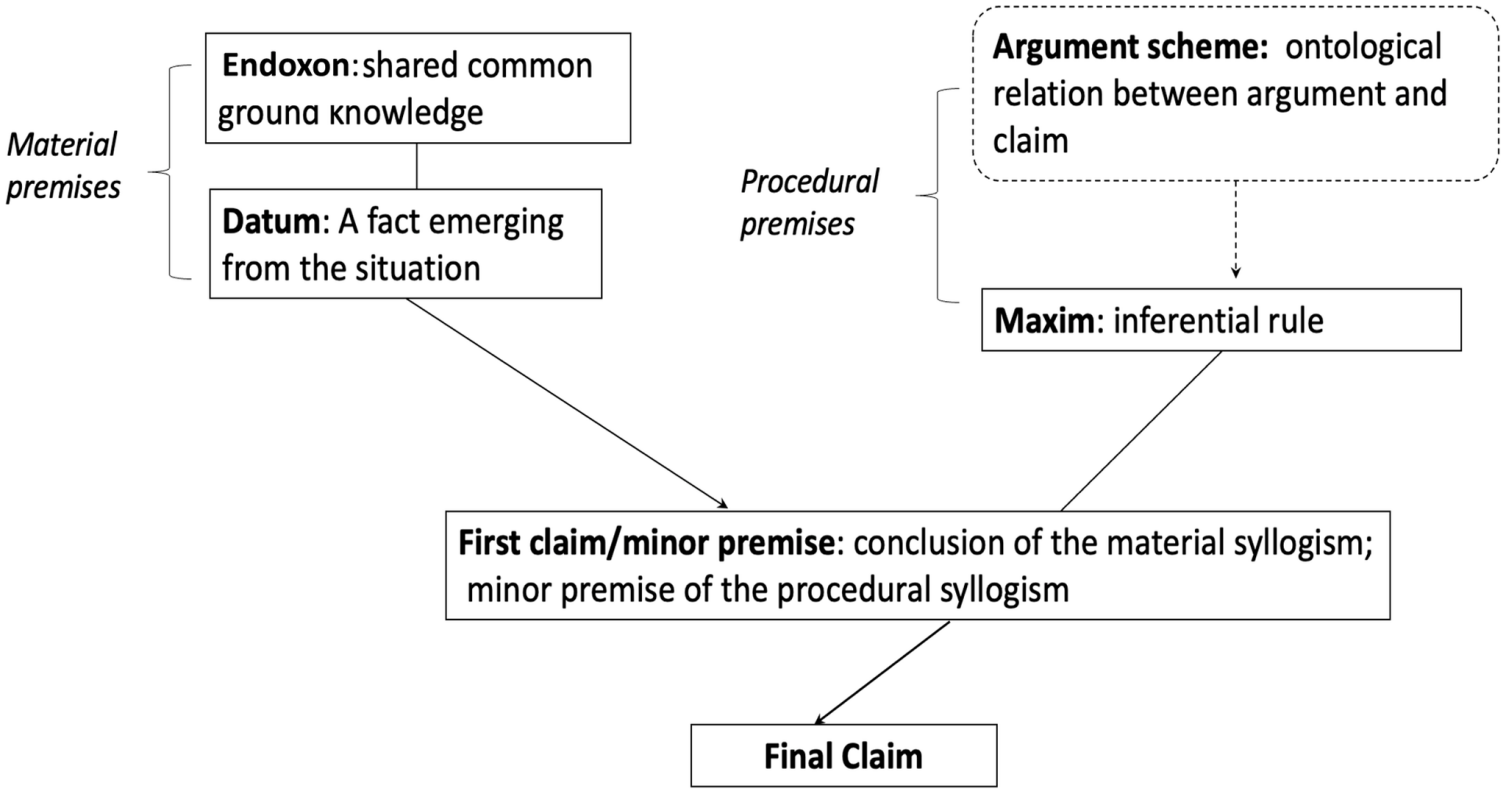
AMT scheme.

To this goal, we propose to apply the argumentum model of topics (AMT; Rigotti and Greco Morasso, 2010; Musi et al., 2016). This is a semantic and pragmatic theory that unpacks the way we draw inferences through argumentative discourse. An argument scheme is the reasoning which “allows moving from the argument premises to its conclusion, or, conversely but equivalently, from the standpoint to the argument supporting it” (Rigotti and Greco Morasso, 2010, p. 491). This approach views an inference as a combination of material and procedural premises. The material premises include the *datum*, which is a piece of textually expressed factual information, and the *endoxon*, which is a proposition referring to beliefs, principles, or values shared within the relevant communicative context (common ground knowledge). The procedural premise is a maxim, an abstract rule of reasoning derived from the argument scheme, which is a general type of relation—such as causality, analogy, or authority—between the argument presented and the claims made. The combination of these two sets of premises allows drawing a conclusion. Figure 1 provides a representation of this scheme.

Consider an everyday claim such as “I cannot bake a strudel because we do not have apples.” The final claim is “I cannot bake a strudel,” while the expressed argument constitutes the *datum* (“We do not have apples”). Through common knowledge, we understand that apples are a necessary ingredient for baking a strudel. Therefore, the argument scheme is causal.

In a fact-checking report, the fact-checker’s verdict (e.g., “news x is fake”) would correspond to the final claim, while the argument scheme would be by definition based on features that allow classifying a news fake according to the sources of information and our common ground knowledge about what counts as a true news. Regardless of the type of decision-making considered in the fact-checking process, our second hypothesis is as follows:

*H2. The distinction between cognitive biases triggered by relevance by effort and those triggered by relevance by effect can be surfaced by reconstructing the inferential pathway of the decision-making process.*

The identification of both the mechanism and the level at which these two types of cognitive biases intervene can provide insights into potential debiasing interventions.

## 4 From theory to practice: a case study with news media practitioners

### 4.1 Method

In our case study, we employed a three-tiered methodology. Initially, we considered the task involved in fact-checking as a type of Information Seeking and Retrieval (ISR). We conducted an assessment to identify ISR-linked cognitive biases relevant to the fact-checking process. To ensure comprehensiveness, we semi-automatically scoped scholarly literature to identify cognitive biases associated with news-making, fact-checking, and journalism which all inherently entail news verification practices. We unified these cognitive biases into a “bias cheat sheet” (Section 4.2). We then categorized our list of biases as “effort related” or “effect related” based on relevance theory (Section 4.3).

**Figure 2:**
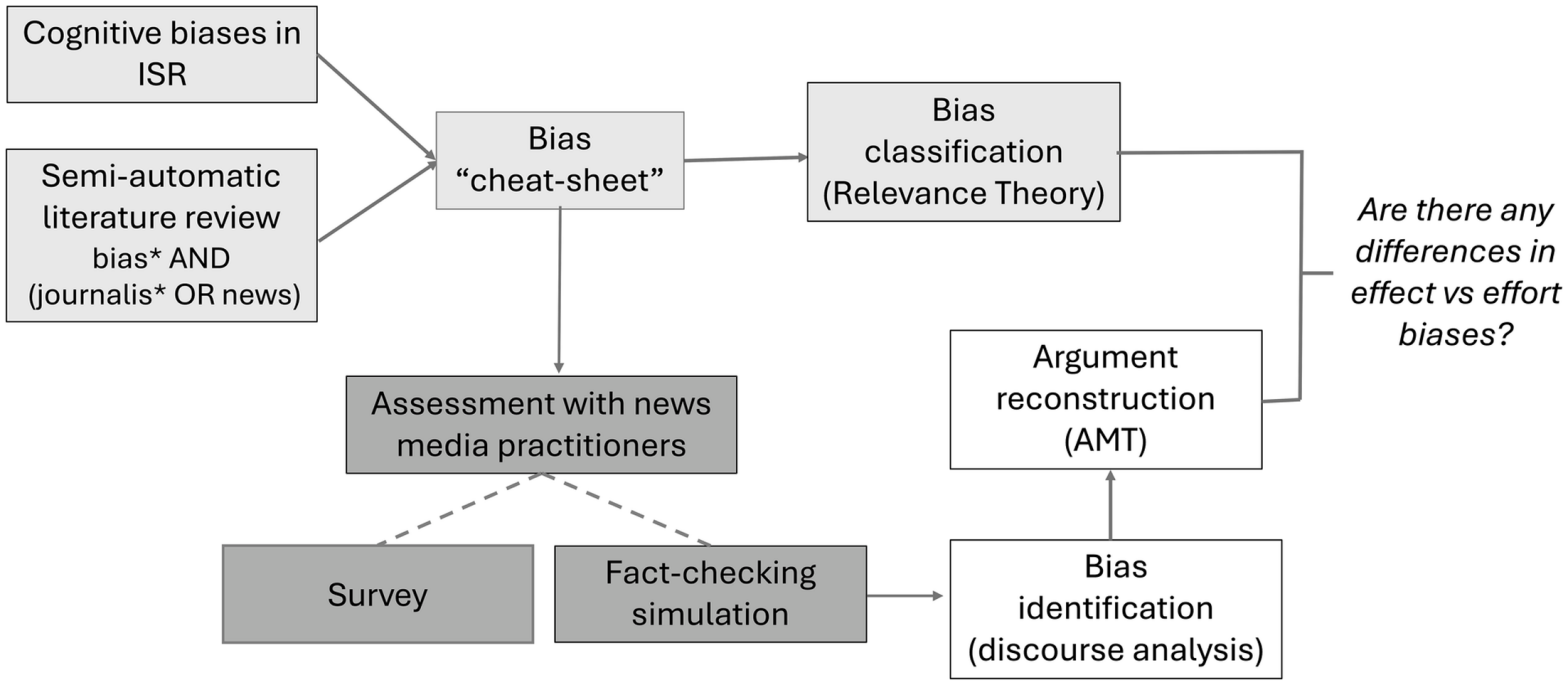
Method overview.

To bridge the gap between theory and real-world scenarios, we conducted interviews with 12 practitioners to evaluate our taxonomy. This involved simulating the fact-checking process and engaging the practitioners in a *post-hoc* discussion (Section 4.4). We adopted the “think aloud” method (Eccles and Arsal, 2017) to track their decision-making process. We then analyzed the transcripts to: (i) identify biases through discourse analysis; and (ii) reconstruct the argument schemes in justifications associated with a potential bias following the argumentum model of topics (Section 4.5). Finally, to corroborate our results, we conducted a survey with 40 practitioners asking them which biases were more likely to influence the fact-checking process (Figure 2).

### 4.2 Cognitive biases identification according to scholarly articles

The first step of our investigation was identifying the subset of cognitive biases affecting the fact-checking process as an ISR task. According to Ellis (1989), Ellis et al. (1993), and Järvelin and Wilson (2003), eight distinct behaviors are fundamental to ISR: starting; browsing; chaining; differentiating; monitoring; extracting; verifying; and ending. When adapted to news verification in a digital setting, these behaviors apply to fact-checking, especially to the stage of evidence retrieval. In fact, ‘Starting’ represents how individuals initiate their search. They may query a search engine, while ‘Browsing’ involves searches to explore information. ‘Chaining’ includes following footnotes, citations, or links for deeper insights, and ‘Differentiating’ filters information based on credibility and relevance. ‘Monitoring’ ensures ongoing awareness through regular searches, and ‘Extracting’ involves selectively retrieving relevant material. ‘Verifying’ rigorously confirms information accuracy, and ‘Ending’ concludes the search process by addressing any remaining questions or details. As a first step, we considered 14 cognitive biases reported by Azzopardi (2021) in their recent review about biases in ISR as preliminary candidates for our taxonomy. From the list, we excluded the following six, either because they are not applicable to professional fact-checking, or for the difficulty in discerning them in real-case scenarios (see Table 1 for the list of those we included):

1. Dunning-Kruger Effect: Tendency of individuals with low ability or knowledge to overestimate their competence, particularly in tasks where they have limited expertise. It does not apply to professional fact-checkers, due to their training and experience.
2. Decoy Effect: The introduction of a third, less desirable option (the decoy) in a choice set can influence individuals to prefer one of the existing options over another, enhancing the attractiveness of the chosen option by comparison. This effect is commonly seen with three options, whereas fact-checking involves verifying numerous news items and sources, reducing the impact of the decoy effect.
3. Peak-End Rule: People tend to evaluate past experiences based on the highest emotional moment (peak) and on how the experience ended, rather than taking into account the overall experience. This bias impacts attitude toward an event personally experienced, and it does not apply to professional fact-checking.
4. Exposure Effects: Similar to reinforcement effects, exposure effects occur when individuals develop a more positive attitude toward a stimulus as a result of repeated exposure. However, exposure effects point to the impact of the amount of time and the number of exposures individuals have to a stimulus. Distinguishing exposure effects from reinforcement effects can be challenging in real-world scenarios because repeated exposure inherently increases both the time spent and the number of engagements with the stimulus. In this study, we focused on reinforcement effects: pages with search results often display multiple results supporting a similar stance, leading to repetitive exposure and increased engagement time with the information.
5. Order and (6) Priming effects: these two biases pose challenges in differentiating them from anchoring effects within real-world scenarios. These cognitive biases are related to the sequence of presentation and contextual cues. Order effects primarily impact memory and recall based on presentation sequence, where the first piece of information is better recalled. Priming effects, on the other hand, activate specific cognitive pathways; for instance, showing a picture of a cat before asking someone to name an animal primes them to respond more quickly with “cat” compared to an unrelated picture. Anchoring effects establish a reference point that influences subsequent judgments. In this context, we focused on anchoring effects as representative of this category.

**Table 1:** List of biases, definitions, and examples included in the bias cheat sheet.

Bias	Definition	Example
Availability bias^*^	Tendency to overestimate the likelihood of an answer or stance based on how easily it can be retrieved and recalled.	A piece of info appears on your news feed and it is shared on your social media walls, making you assume it is accurate.
Ambiguity effect^*^	Tendency to avoid options in which there is high uncertainty in the outcome, even if they are favorable.	You do not believe that an earthquake is gonna happen as scientific evidence cannot predict it with certainty higher than 60%.
Anchoring bias^*^	Tendency to focus too much on the first piece of information learned, or observed, even if that information is not relevant or correct.	The first entry emerging from a Google search is deemed more important than the entries that follow, regardless of their relevance.
Bandwagon effect^*^	Tendency to take on a similar opinion or point of view because other people voice that opinion or point of view.	A tweet went viral; thus, it must convey true content.
Confirmation bias^*^	Tendency to prefer confirmatory information and to discount information that does not conform to existing beliefs.	You trust evidence that points to despicable behavior of a politician belonging to a party you would never vote for.
Framing effect^*^	Tendency to make different decisions given the same information because of how the information has been presented.	The investments of a company in net-carbon are presented as green-washing without touching upon positive outcomes.
Less-is-more effect^*^	When many options are presented, people find it harder to make comparisons and often will not make any decisions.	You feel overwhelmed by the amount of info about ChatGPT and you decide to avoid reading any news about it.
Reinforcement effect^*^	Tendency to develop a positive attitude toward a stimulus if it is seen multiple times.	You believe that a celebrity is dead because the search results page shows many results purporting it.
Selection bias	How events are either selected into the media record or left unreported.	Out of 10 polls, the results of three are reported and the other ones are neglected.
Source bias	The selection of who is to speak for or about an event, how and where they are filmed, and what they are asked to and allowed to say.	You rely solely on news from the New York Times to report on American polls results.

* From Azzopardi (2021).

As a second step, we then broadened our examination to encompass cognitive biases impacting the (dis-)information environment identified in the scholarly literature. We utilized the Scopus API to automatically retrieve all scientific papers published since 1996 that explicitly mentioned the term “bias*” AND (journals* OR news). From the 3,407 retrieved abstracts, we extracted all bigrams containing the terms “bias” or “effect.” A bigram is a sequence of two adjacent items, such as words or tokens, within a text or dataset. Looking at the bigrams, we identified two other biases previously not included in the list of Azzopardi’s (2021). We thus consolidated our list and associated each bias with a definition and an illustrative example applicable to the news environment. This process resulted in our “bias cheat sheet” (Table 1).

### 4.3 Cognitive biases classification according to relevance theory (H1)

We classified the biases described in Section 4.2 based on whether they are primarily associated with ‘relevance by effort’ or ‘relevance by effect’. By “primarily,” we mean that we recognize that the two aspects are not mutually exclusive, but one aspect is more prominent. For ‘relevance by effort’ biases, we included those that reduce cognitive effort in processing information, as they demand less effort in terms of perception, memory, or inference. As a result, such information is more deserving of our attention because the processing is perceived as more rewarding. In the fact-checking context, these biases are likely to affect evidence retrieval:

- Availability bias: Information stored in memory with high accessibility or availability can be retrieved rapidly and with minimal effort. When information is easily retrievable, it requires fewer cognitive resources for access and processing, leading to reduced cognitive load (e.g., Barrouillet et al., 2007).
- Anchoring bias: adjustments after having been presented with a piece of information require cognitive effort (e.g., Epley and Gilovich, 2006).
- Framing effect (images): pieces of information can be framed through images. Visual stimuli and patterns are elaborated quickly and without a significant conscious effort (e.g., Huff et al., 2018).
- Less-is-more effect: a great number and complexity of information leads to information overload, which can exceed information processing capacities (e.g., Che et al., 2019).
- Reinforcement effect: repetition leads to reactivation of stimuli in working memory (Şentürk et al., 2024). As for availability bias, this leads to reduced cognitive load for processing it.

We categorized the other biases as ‘relevance by effect’, instead, as they are activated when interpreting new information which updates the individual’s representation of the world (e.g., verifying that a piece of info is true). Unlike relevance biases driven by effort, these biases operate on an epistemological level, influencing the interpretation of incoming information through the lens of existing beliefs and background knowledge. In fact, background knowledge serves as the context through which new information is understood, leading to conclusions deemed most relevant. As a result, information that aligns with pre-existing individual/shared beliefs and that is presented through clear and/or known templates will be seen as more relevant during the news verification process, helping to maintain a consistent worldview:

- Ambiguity effect: People generally seek certainty and are drawn to options that offer clear outcomes (e.g., Chatterjee et al., 2014). Uncertain pieces of information are less likely to prompt updates to their beliefs.
- Bandwagon effect: Humans have a natural tendency to conform to group norms and seek social acceptance. Adopting a popular opinion can align with these social dynamics so that a change in understanding in a popular direction can result in desirable outcomes (Farjam and Loxbo, 2023).
- Confirmation bias: Accepting information that aligns with our beliefs minimizes cognitive dissonance—the discomfort or tension that arises from holding conflicting beliefs. Updating our worldview with consistent information helps alleviate this discomfort (Knobloch-Westerwick et al., 2020).
- Framing effect (textual): depending on how the information is framed, its salience is different (Van Schie and Van Der Pligt, 1995); consequently, the same piece of information will result as more or less relevant according to how it is presented.
- Selection bias: media organizations can select events or pieces of information for achieving newsworthiness (Lundman, 2003) leading to a greater cognitive effect—and consequently relevance—for their audience.
- Source bias: trusted sources are more likely to have an impact on the updating of individuals’ world views (Wallace et al., 2020).

### 4.4 Mapping cognitive biases according to argumentum model of topics (H2)

#### 4.4.1 The fact-checking simulation

We conducted a focus group and six individual interviews with practitioners experienced in news-making and fact-checking. Participants (*N* = 12, including 3 journalists and 9 fact-checkers) were recruited through the ‘network of research team. To ensure diverse perspectives and representation of various experiences, participants varied in affiliation, age, gender, country where their organization is based, and years of work in the field. The focus group was conducted face-to-face and lasted for 2 h. It was audio-recorded and transcribed. The individual interview sessions, each lasting 90 min, were conducted online, video-recorded, and transcribed. The study received ethical approval from the University of Liverpool ethics committee (ref. N. 12,705 and 12,688).

The activity was divided into two parts. In the first part, participants engaged in a fact-checking simulation. To keep track of their reasoning during the tasks, we used the think-aloud technique (Ericsson and Simon, 1993; e.g., Güss, 2018). This method asks respondents to verbalize their thoughts and reasoning while performing a task. This technique provides insight into cognitive processes and decision-making strategies. We chose this approach for two reasons. First, the final stage of fact-checking is explicated by the fact-checking report, but the underlying reasons for news selection and evidence retrieval are implicit and call for explanations. Second, accessing the reasoning in real-time as it unfolds helps avoid relying solely on *post-hoc* justifications, offering a more accurate understanding of the decision-making process. Throughout all phases, participants were also given specific time constraints to encourage prioritization strategies.

The fact-checking simulation was divided into three phases: news selection; evidence retrieval; and fact-check report writing.

- News selection. During this phase, participants were presented with eight fictitious news articles (Supplementary materials) related to artificial intelligence (AI) or climate change and were asked to select one for fact-checking during the simulation. The initial drafts of the articles were generated using GPT-3 and then edited to create their final versions. These articles were designed to reflect the diversity of the (mis)information ecosystem, varying in sources, the inclusion of multimedia features (with four of eight containing images), and truth values. Specifically, we included two true news articles, two fake news articles, and four articles containing common tactics of misinformation, such as viral advertisement, misattribution, cherry-picking, misleading images, and correlation-not-causation (Musi et al., 2023). At the conclusion of this phase, participants were asked to explain the three main reasons behind their choice of a specific news article.
- Evidence retrieval. This phase focused on information seeking and retrieval. Participants were instructed to approach the task as they would in their typical work routine and find information to fact-check the selected news article. They were encouraged to take notes in their preferred format.
- Fact-check report writing. Finally, we asked our participants to provide a score on the fakeness of the news on a 5-point Likert scale (from “Strongly disagree” to “Strongly agree”) and to write a fact-check report composed of three paragraphs and a title to support their rating.

Following the simulation, participants were asked the question: “What are the main differences and similarities between how you approached the activity today versus how you would typically approach it in your daily work?” to ensure the ecological validity of our simulation. Finally, participants were presented with our ‘bias cheat sheet’ and asked to identify the two biases they deemed most relevant to fact-checking practices. Participants stated that—apart from the time constraints given—during the fact-checking process they behaved as they would do during their day-to-day working life.

#### 4.4.2 Mapping the cognitive biases

Guided by the bias cheat sheet, we identified in the transcripts all the passages indicating the intervention of a cognitive bias (*datum*). As presented in Section 4.1, the transcript captured participants’ reasoning during the fact-checking simulation, recorded through the think-aloud technique. The authors were involved in all the steps of the analysis. First, one author read each transcript and, using the definitions provided in the bias cheat sheet, highlighted all passages where reasoning aligned with one of the listed biases. Each relevant segment was categorized with the corresponding bias name. For example, the statement (*datum*), “[I chose News 4 because] it reminds me of other recent fires due to climate change” was marked as an instance of availability bias (the tendency to overestimate the likelihood of an answer or stance based on how easily it can be recalled). In this case, the news topic was readily accessible in the participant’s memory and was reported as a main argument to support their choice. The other authors read each transcript and reviewed the first author’s study, discussing potential cases of disagreement. The only instance of disagreement involved distinguishing between confirmation bias and source bias. The authors recognized that source bias can presuppose a confirmation bias justifying the evaluation of sources as trustworthy or adequate. It was thus decided to prioritize source bias as the bias directly impacting decisions and keep confirmation bias, for instance, where source bias was ruled out.

**Figure 3:**
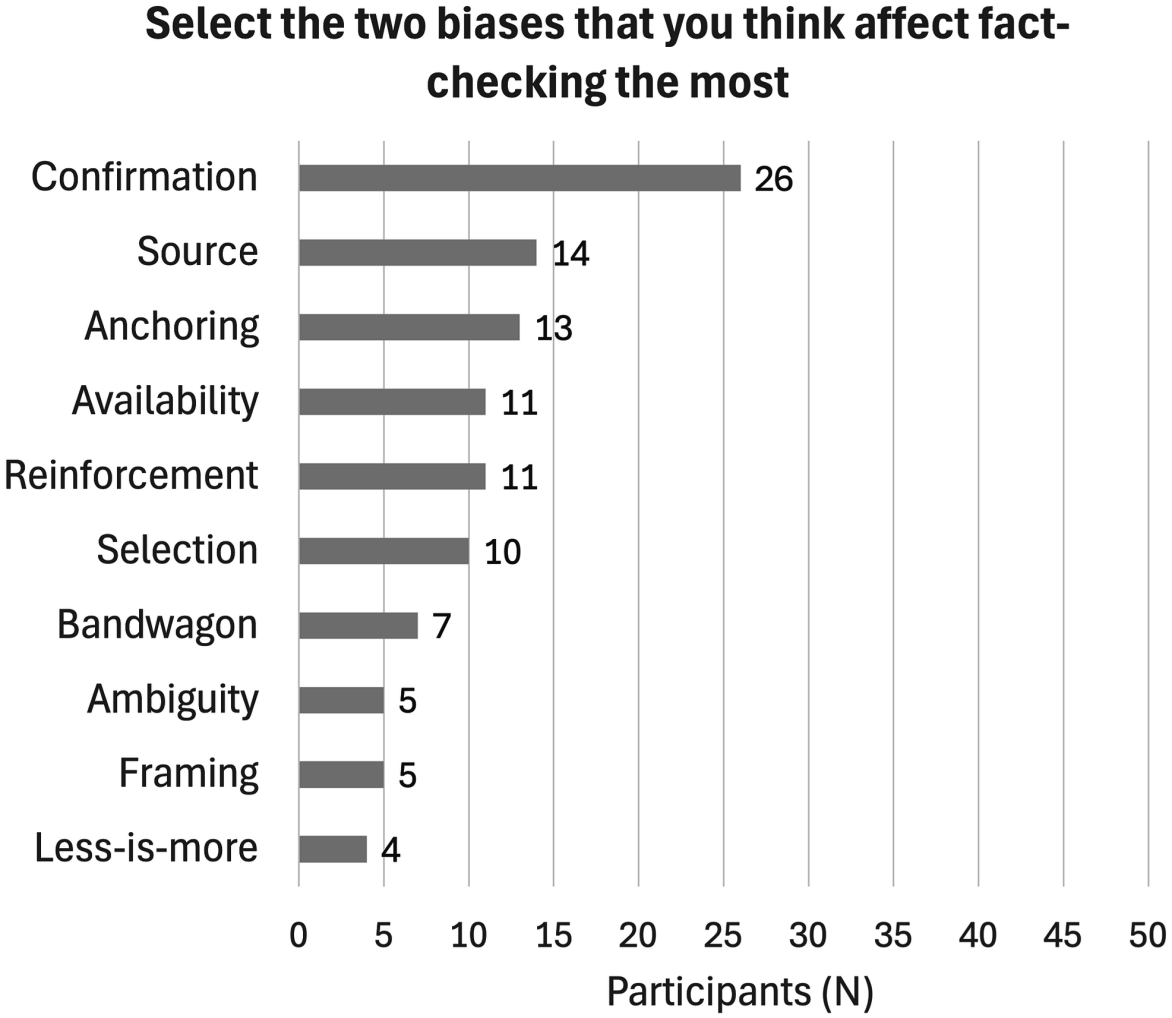
Biases selected by the practitioners.

From the transcripts, we observed six out of the 10 biases we initially identified (reinforcement effect, anchoring, availability, confirmation, source, and selection biases). According to our classification (see Section 4.3), both the categories ‘relevance by effort’ and ‘relevance by effect’ were represented. As per our hypothesis, both biases triggered by ‘relevance by effort’ and ‘relevance by effect’ can intervene in the fact-checking process. In particular, these same biases were consistently selected as the most influential on the fact-checking process when participants were explicitly asked their professional opinions after the simulation. However, the interviews included a limited number of media practitioners. To determine whether this *post-hoc* perception is specific to the interview group or is likely shared by other professionals, we complemented the interviews with an online survey. Figure 3 shows the answers collected during the focus groups, interviews, and survey including a total of 52 participants, comprising 36 journalists, 12 fact-checkers, and 4 individuals with experience in both roles, from organizations based in Austria, Hungary, Italy, the UK, Romania, Slovenia, and Spain. The results confirmed the overlap between the biases we observed during the simulation and those perceived as most relevant for the fact-checking process.

**Figure 4:**
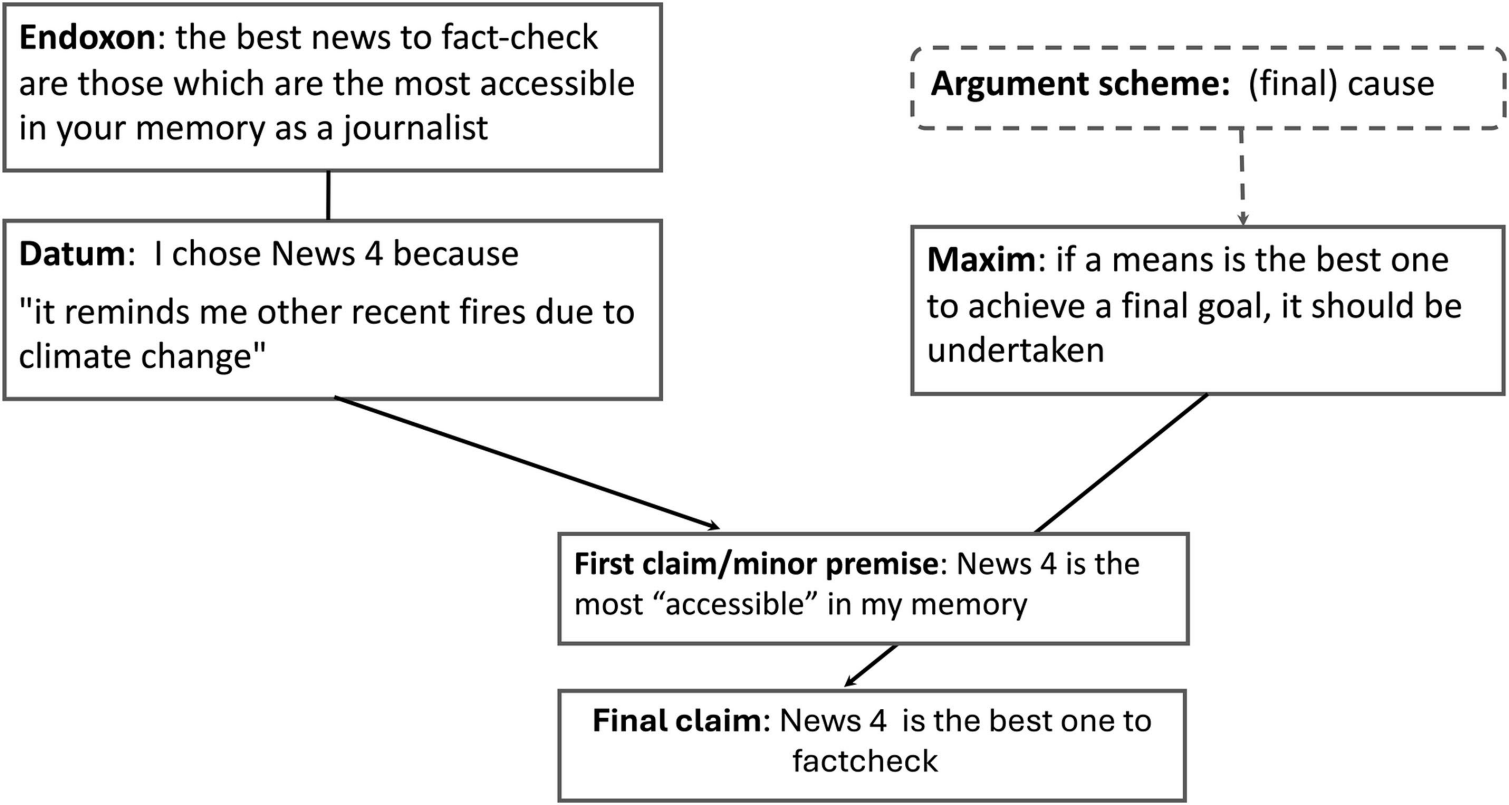
Example of argument scheme connected to availability bias (relevance by effort).

**Figure 5:**
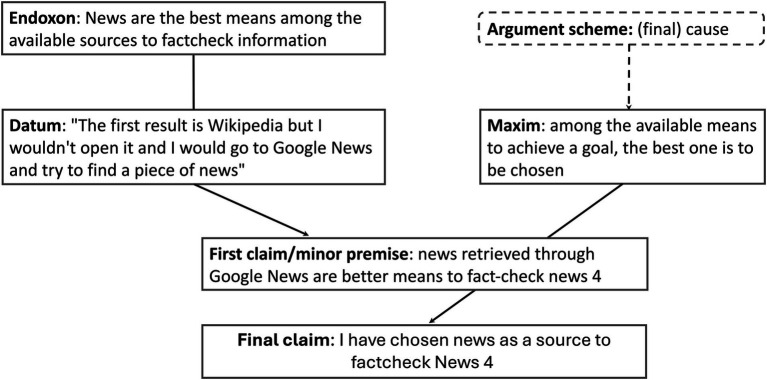
Example of argument scheme connected to source bias (relevance by effect).

Once all the passages related to cognitive biases were categorized, starting from the *datum* identified in the transcripts, we reconstructed the argument scheme following the scheme presented in Section 3.3 (Figure 4 shows an example of relevance by effort bias—availability bias; Figure 5 for relevance by effect bias—source bias), where the *datum* corresponds to the argument explicitly provided by the professional to justify their choice of news/piece of evidence/news rating (final claim), while the *endoxon* is the piece of common knowledge that allows us to interpret the *datum* as an argument for the final claim. The argument scheme is the reasoning that allows to the connection of the *datum* and the *endoxon* to the final claim. In our case, the types of argument schemes were constrained by the structure of the simulation experience: while, as explained in section 3.3., the reasoning behind the final verdict in a fact-checking report is definitional, the argument schemes used to justify the selection of news to fact-check or evidence to consider are causal. These schemes pinpoint the most effective means—such as highly popular news stories or significant pieces of evidence—to achieve the goals of preventing the spread of dangerous fake news and selecting the most relevant evidence to assess the veracity of the news.

We applied this approach for each identified cognitive bias. We observed as hypothesized that a difference emerged among the two types of biases. In particular, we could map this difference in the *endoxa* (common ground knowledge) among biases triggered by relevance through effort versus those influenced by relevance through effect.

Table 2 presents the biases categorized as “effort related” that emerged during the simulation, along with an example of the *datum* and associated *endoxon* (common ground knowledge).

**Table 2:** Effort-related biases with an example of *datum* and reconstructed *endoxon* (common ground knowledge).

Bias	*Datum* (examples)	*Endoxon* (examples)
Anchoring bias	“[I unconsciously chose News 3 because] the news which was displayed when I went back to the tab with all the news, that was the one which was displayed in front of me.”	The best news to fact-check for a fact-checker are those which are the first seen.
Availability bias	“[I chose News 4 because] it reminds me other recent fires due to the climate change.”	The best news to fact-check for a journalist are those which are the most available in their memories.
Reinforcement bias	“[News 6 is a fake news because] the hashtag RespectCreativity got corrected by the Google browser into #RespectCreaEvity and the hashtag #SupportArtists come out as #SupportArEsts multiple times.”	Hashtags that get corrected multiple times are fabricated (spelling-wise).

The shared knowledge underlying the data in the case of effort-triggered biases suggests that the decision-making strategy employed not only is suboptimal for achieving accurate results but also that it is linked to contextual factors of which fact-checkers are not aware at the time of the choice. Consequently, making the endoxical reasoning explicit might directly prompt the practitioner to reconsider their choices. For example, a participant acknowledging the anchoring effect commented that they were not aware of the reasons underlying their choice when they selected that piece of news. Regarding the reinforcement effect, it is notable that the hashtag was not corrected on other devices.

Conversely, participants seem to be aware of the *endoxa* (common ground knowledge) associated with relevance by effect, and also, the reasoning might appear more sounding to them (Table 3).

**Table 3:** Effect-related biases with an example of *datum* and reconstructed *endoxon* (common ground knowledge).

Bias	*Datum* (examples)	*Endoxon* (examples)
Confirmation bias	“About this part of the news, I heard about this. So I already know that this is mostly true. I think I should fact check my conviction, but it’s true that. I mean, it’s it seems to me that it’s true. It’s also quite logical. […] okay due to the time constraint I will not fact-check more this part I’m pretty sure this is true but still very partial.”	Information accessed by hearsay which sounds reasonable is not the best to prioritize in an effective fact-check.
Sourcebias	“The first result is Wikipedia, but I would not open it and I would go to Google News and try to find a piece of news.”	News are the best means among the available to fact-check information.
Selection bias	The claim on the Northway passage in News 5 has not been fact-checked and reported in the fact-checking report.	It is not necessary to report the claims that have not been fact-check in the fact-check report.

While these *endoxa* (common ground knowledge) might appear reasonable to the participants and they are conscious about the reasoning grounding their choice, they do not align with best practices outlined by the International Fact-Checking Network. According to these standards, all relevant claims should be fact-checked, prioritizing first-hand sources when available (e.g., reports, data, and witnesses), and providing explanations for the choice of claims to be checked whenever possible. In these cases, different debiasing strategies might be more effective (e.g., recalling the IFCN standards).

## 5 Discussion

The advent of digitization has created a fast-paced and densely populated information landscape where distinguishing information from mis- and disinformation is a critical issue. Fact-checkers play an effective role in fighting fake news, but they also face challenges imposed by the (dis-)information ecosystem. The continuous stream of news increases cognitive load and time pressure, factors recognized as making cognitive biases more likely. While scholars have focused on cognitive biases influencing audience decisions, little attention has been given to how biases can influence the professional news verification process. Nonetheless, developing standards to mitigate them can enhance the quality of fact-checks while reinforcing audiences’ trust and preventing reputation attacks from disinformation actors. To achieve this goal, it is paramount to identify: (i) what biases are the most frequent in the context of news verification; (ii) what are their triggers; and (iii) at what level of the reasoning involved in news verification they act. As to (i), we have compiled a set of biases relevant to the fact-checking process through a semi-automatic literature review and we validate it with practitioners. To tackle (ii) and (iii), we propose a theoretically grounded methodology to do so. We conceive fact-checking as an argumentative process whereby the fact-checker must advance an evaluative standpoint (“news X is true/false”) supported by arguments retrieved through information verification. While the final verdict is argued for in the fact-check, the process of verification requires at least two further steps, implicit for the audience, of news selection, and of sources selection. Drawing upon relevance theory, we argue that relevance plays a crucial role in guiding each of the decision-making processes based on both individual beliefs and contextual factors. This allows us to classify the biases into two broader groups—triggered by relevance by effect and by relevance by effort (H1). We then further hypothesize that the difference between these two groups of biases can be surfaced reconstructing the reasoning processes involved in the decision-making through the argumentum model of topics (H2).

To test the explanatory potential of our framework in a real-world setting, we conducted a fact-checking simulation with professional fact-checkers. We observed the biases intervening during the news selection, evidence retrieval, and in the fact-check report writing phases; we then reconstructed the associated reasoning paths (argument schemes) according to the argumentum model of topics. Our study supports the explanatory potential of the framework: first, during the simulation, both biases induced by relevance by cognitive efforts and relevance by cognitive effects are observed (H1). Second, we notice a difference emerging in the types of common grounds (*endoxa*) associated with the two classes of biases: *endoxa* associated with cognitive biases by effect is grounded on fact-checkers’ actual beliefs (e.g., the untrustworthiness of sources coming from x), while those associated to biases by effort reflect empirical shortcuts (e.g., it is easier to fact-check familiar news), which fact-checkers are not necessarily aware of undertaking (H2).

The framework we introduced helps identify cognitive biases in the fact-checking process and clarify their underlying triggers. Differentiating the two categories of bias triggers—relevance by effort and relevance by effect—has practical implications for debiasing strategies. For biases by effort, reconstructing the reasoning path can effectively counteract their influence. For example, if fact-checkers realize they chose to verify a piece of news simply because it was the first they encountered (anchoring bias), the inappropriateness of such a criterion becomes apparent. Interventions prompting fact-checkers to decompose their reasoning and reflect upon the *endoxa* (common background) underlying their decisions can work as effective debiasing measures. Similarly, showing the reconstructions of such reasonings helps locate the roots of the bias that is frequently triggered by digital media affordances and their recommendation systems.

Biases by effect are more challenging to counter when they involve common background knowledge, as these biases operate on an epistemological level. For example, demonstrating that all checked sources are from news articles (rather than, say, scientific reports) may not reduce source bias if the fact-checker considers these sources to be the most reliable. However, such a finding would raise doubts if it directly conflicts with IFCN guidelines. In other words, analyzing the triggers of biases by effect should inform IFCN guidelines, bridging abstract principles with their practical application.

Furthermore, the classification we propose is essential for designing digital tools that support fact-checkers’ decision-making processes. Based on our findings, we have developed an online tool that incorporates the analysis of competing hypotheses (ACH)—a structured analytic technique originally developed by the Central Intelligence Agency to mitigate biased decision-making among intelligence analysts (Heuer, 1999)—and adapted it to the fact-checking domain (freely accessible at: arg.tech/latif-app). The distinction between biases by effort and biases by effect has directly informed the types of interventions offered by the tool. For example, biases by effort are addressed through tabular visualizations and automatic assistance in identifying claims within a source, while biases by effect are countered through strategies like warnings that nudge users to reflect more critically.

Our contribution focuses on third-party fact-checking, which targets news articles that have already been published, but it also has broader applicability to journalistic practices. It is particularly relevant to internal fact-checking, which involves verifying information and claims in news articles before publication. However, biases related to effort are relevant to many stages of journalistic work—ranging from editorial decisions to reporting—when selecting a story to cover, gathering sources, and choosing the language to frame the story. In other words, such biases are present whenever information is gathered online, regardless of whether the goal is news reporting or fact-checking, as they are entrenched and amplified by digital affordances. When it comes to biases by effect the agenda setting of different news-making outlets might play a role, while non-partisanship is a foundational requirement for fact-checking (e.g., Principle #1 of the International Fact-Checking Network: ‘A commitment to Non-partisanship and Fairness’; https://ifcncodeofprinciples.poynter.org/the-commitments). Nevertheless, the principle of impartiality which underpins main news media outlets shows that the mitigation of both types of biases is an ideal that goes beyond the fact-checking domain.

We acknowledge that our list of potential cognitive biases affecting professional fact-checking was not exhaustive. In addition, our sample size during interviews was limited due to our focus on engaging news media practitioners in a quasi-ecological activity. Providing a comprehensive list was beyond the aim of this contribution but future studies could broaden the sample size and delve deeper into the explanatory capabilities of our approach, proposing different news articles to elicit a broader variety of cognitive biases and further exploring whether other differences exist. We also think that it would be valuable to systematically validate how the identified *endoxa* are perceived by the practitioners and explore the effect that this awareness might have on their final decisions.

Overall, our study contributes to a better understanding of cognitive biases in the news landscape. From a theoretical point of view, we offer the first empirically based taxonomy of cognitive biases incurring in fact-checking. This taxonomy promises to be relevant for news verification in general, beyond the fact-checking professional domain. From a methodological point of view, we develop an interdisciplinary framework that allows us to map at what stage of the decision-making process different classes of bias act. From a practical point of view, recognizing the difference between biases triggered by effort and those triggered by effect and their inferential role can inform debiasing strategies. Fact-checkers can, in fact, reflect upon their reasoning paths becoming aware of how behaviors induced by the networked society might not always align with best practices and becoming resilient toward cognitive biases.

## Data Availability

The raw data supporting the conclusions of this article will be made available by the authors, without undue reservation.

## References

[ref1] Allred S. R. Crawford L. E. Duffy S. Smith J. (2016). Working memory and spatial judgments: cognitive load increases the central tendency bias. Psychon. Bull. Rev. 23, 1825–1831. doi: 10.3758/s13423-016-1039-0, PMID: 27084778

[ref2] Amazeen M. A. (2013). A critical assessment of fact-checking in 2012. Washington, DC: New America Foundation.

[ref3] Amazeen M. A. (2015). Revisiting the epistemology of fact-checking. Crit. Rev. 27, 1–22. doi: 10.1080/08913811.2014.993890

[ref4] Azzopardi L. (2021). “Cognitive biases in search: a review and reflection of cognitive biases in information retrieval.” in *Proceedings of the 2021 Conference on Human Information Interaction and Retrieval*. pp. 27–37.

[ref5] Barrouillet P. Bernardin S. Portrat S. Vergauwe E. Camos V. (2007). Time and cognitive load in working memory. J. Exp. Psychol. Learn. Mem. Cogn. 33:570. doi: 10.1037/0278-7393.33.3.570, PMID: 17470006

[ref6] Bauer C. A. Hannover B. (2020). Changing “us” and hostility towards “them”—implicit theories of national identity determine prejudice and participation rates in an anti-immigrant petition. Eur. J. Soc. Psychol. 50, 810–826. doi: 10.1002/ejsp.2666

[ref7] Benson B. (2016). Cognitive bias cheat sheet. Better Humans, September. Available at: https://betterhumans.coach.me/cognitive-bias-cheat-sheet-55a472476b18 (Accessed November 25, 2024).

[ref8] Berthet V. (2022). The impact of cognitive biases on professionals’ decision-making: a review of four occupational areas. Front. Psychol. 12:802439. doi: 10.3389/fpsyg.2021.802439, PMID: 35058862 PMC8763848

[ref9] Carnahan D. Bergan D. E. (2022). Correcting the misinformed: the effectiveness of fact-checking messages in changing false beliefs. Polit. Commun. 39, 166–183. doi: 10.1080/10584609.2021.1963358

[ref10] Chatterjee S. Atav G. Min J. Taylor D. (2014). Choosing the sure gain and the sure loss: uncertainty avoidance and the reflection effect. J. Consum. Mark. 31, 351–359. doi: 10.1108/JCM-04-2014-0949

[ref11] Che J. Sun H. Xiao C. Li A. (2019). Why information overload damages decisions? An explanation based on limited cognitive resources. Adv. Psychol. Sci. 27, 1758–1768. doi: 10.3724/SP.J.1042.2019.01758

[ref12] Croce M. Piazza T. (2023). Consuming fake news: can we do any better? Soc. Epistemol. 37, 232–241. doi: 10.1080/02691728.2021.1949643, PMID: 39447166

[ref13] Davison W. P. (1983). The third-person effect in communication. Public Opin. Q. 47, 1–15. doi: 10.1086/268763, PMID: 39496072

[ref14] Dimara E. Franconeri S. Plaisant C. Bezerianos A. Dragicevic P. (2018). A task-based taxonomy of cognitive biases for information visualization. IEEE Trans. Vis. Comput. Graph. 26, 1413–1432. doi: 10.1109/TVCG.2018.2872577, PMID: 30281459

[ref15] Eccles D. W. Arsal G. (2017). The think aloud method: what is it and how do I use it? Qual. Res. Sport, Exerc. Health 9, 514–531. doi: 10.1080/2159676X.2017.1331501, PMID: 39560832

[ref16] Ellis D. (1989). A behavioural approach to information retrieval design. J. Doc. 45, 171–212.

[ref17] Ellis D. Cox D. Hall K. (1993). A comparison of the information seeking patterns of researchers in the physical and social sciences. J. Doc. 45, 171–212. doi: 10.1108/eb026843

[ref18] Ellsberg D. (1961). Risk, ambiguity, and the savage axioms. Q. J. Econ. 75, 643–669. doi: 10.2307/1884324

[ref19] Endsley M. R. (2018). Combating information attacks in the age of the internet: new challenges for cognitive engineering. Hum. Factors 60, 1081–1094. doi: 10.1177/0018720818807357, PMID: 30376429

[ref20] Epley N. Gilovich T. (2006). The anchoring-and-adjustment heuristic: why the adjustments are insufficient. Psychol. Sci. 17, 311–318. doi: 10.1111/j.1467-9280.2006.01704.x, PMID: 16623688

[ref21] Ericsson K. A. Simon H. A. (1993). Protocol analysis: Verbal reports as data. Rev. Edn. Cambridge, MA: Bradford Books/ MIT Press.

[ref22] European Parliament (2023). TV still main source for news but social media is gaining ground. Available at: https://www.europarl.europa.eu/news/en/press-room/20231115IPR11303/tv-still-main-source-for-news-but-social-media-is-gaining-ground (Accessed November 25, 2024).

[ref23] Farjam M. Loxbo K. (2023). Social conformity or attitude persistence? The bandwagon effect and the spiral of silence in a polarized context. J. Elect. Public Opin. Parties. 34, 1–21. doi: 10.1080/17457289.2023.2189730

[ref24] French A. M. Storey V. C. Wallace L. (2023). The impact of cognitive biases on the believability of fake news. Eur. J. Inf. Syst., 1–22. doi: 10.1080/0960085X.2023.2272608, PMID: 38075016

[ref25] Graves L. Amazeen M. (2019). Fact-checking as idea and practice in journalism. Oxford Research Encyclopedia of Communication. Oxford, UK: Oxford University Press.

[ref26] Güss C. D. (2018). What is going through your mind? Thinking aloud as a method in cross-cultural psychology. Front. Psychol. 9:1292. doi: 10.3389/fpsyg.2018.01292, PMID: 30150948 PMC6099082

[ref27] Hasher L. Goldstein D. Toppino T. (1977). Frequency and the conference of referential validity. J. Verbal Learn. Verbal Behav. 16, 107–112. doi: 10.1016/S0022-5371(77)80012-1, PMID: 27863724

[ref28] Heuer R. J. (1999). Psychology of intelligence analysis. Langley, Virginia: Center for the Study of Intelligence.

[ref29] Huff T. Mahabadi N. Tadi P. (2018). Neuroanatomy, visual cortex. Treasure Island, FL: StatPearls Publishing. 29494110

[ref30] International Fact-Checking Network (2024). IFCN Code of Principles. Available at: https://www.ifcncodeofprinciples.poynter.org/the-commitments (Accessed November 25, 2024).

[ref31] Järvelin K. Wilson T. D. (2003). On conceptual models for information seeking and retrieval research. Inf. Res. 9, 9–1.

[ref32] Jenkins H. (2006). “Confronting the challenges of participatory culture: Media education for the 21st century.” in An occasional paper on digital media and learning. John D. and Catherine T. MacArthur Foundation.

[ref33] Jylhä K. M. Stanley S. K. Ojala M. Clarke E. J. (2022). Science Denial. European Psychologist.

[ref34] Karduni A. Wesslen R. Santhanam S. Cho I. Volkova S. Arendt D.. (2018). “Can you verifi this? Studying uncertainty and decision-making about misinformation using visual analytics.” In *Proceedings of the International AAAI Conference On Web and Social Media (Vol. 12, No. 1)*.

[ref35] Knobloch-Westerwick S. Mothes C. Polavin N. (2020). Confirmation bias, ingroup bias, and negativity bias in selective exposure to political information. Commun. Res. 47, 104–124. doi: 10.1177/0093650217719596

[ref36] Korteling J. E. Toet A. (2020). Cognitive biases. Encyclopedia of behavioral neuroscience.

[ref37] Lichtenstein S. Slovic P. Fischhoff B. Layman M. Combs B. (1978). Judged frequency of lethal events. J. Exp. Psychol. Hum. Learn. Mem. 4:551. doi: 10.1037/0278-7393.4.6.551, PMID: 731196

[ref38] Lindeman M. Svedholm-Häkkinen A. M. Riekki T. J. (2023). Searching for the cognitive basis of anti-vaccination attitudes. Think. Reason. 29, 111–136. doi: 10.1080/13546783.2022.2046158

[ref39] Lundman R. J. (2003). The newsworthiness and selection bias in news about murder: Comparative and relative effects of novelty and race and gender typifications on newspaper coverage of homicide. Sociol. Forum 18, 357–386. doi: 10.1023/A:1025713518156

[ref40] Manoogian J. Benson B. (2017). Cognitive bias codex. Available at: https://betterhumans.coach.me/cognitive-bias-cheat-sheet-55a472476b18 (Accessed November 25, 2024).

[ref41] Martínez-Costa M. P. López-Pan F. Buslón N. Salaverría R. (2023). Nobody-fools-me perception: influence of age and education on overconfidence about spotting disinformation. Journal. Pract. 17, 2084–2102. doi: 10.1080/17512786.2022.2135128

[ref42] Matute H. Blanco F. Yarritu I. Díaz-Lago M. Vadillo M. A. Barberia I. (2015). Illusions of causality: how they bias our everyday thinking and how they could be reduced. Front. Psychol. 6:888. doi: 10.3389/fpsyg.2015.00888 26191014 PMC4488611

[ref43] Milovidov V. (2018). Hearing the sound of the wave: what impedes one’s ability to foresee innovations? Форсайт 12, 88–97. doi: 10.17323/2500-2597.2018.1.88.97

[ref44] Minielli M. C. (2010). ISSA proceedings 2010–“crisis” and argument by definition in the modern American presidency.

[ref45] Monaco A. Kotz J. Al Masri M. Allmeta A. Purnhagen K. P. König L. M. (2024). Consumers’ perception of novel foods and the impact of heuristics and biases: a systematic review. Appetite 196:107285. doi: 10.1016/j.appet.2024.107285, PMID: 38423301

[ref46] Morales-i-Gras J. (2020). Cognitive biases in link sharing behavior and how to get rid of them: evidence from the 2019 Spanish general election twitter conversation. Soc. Media Soc. 6:2056305120928458. doi: 10.1177/2056305120928458

[ref47] Musi E. Carmi E. Reed C. Yates S. O’Halloran K. (2023). Developing misinformation immunity: how to reason-check fallacious news in a human–computer interaction environment. Soc. Media Soc. 9:20563051221150407. doi: 10.1177/20563051221150407

[ref48] Musi E. Ghosh D. Muresan S. (2016). “Towards feasible guidelines for the annotation of argument schemes.” in *Proceedings of the Third Workshop on Argument Mining (ArgMining2016)*. pp. 82–93.

[ref49] Mutz D. C. (1998). Impersonal influence: How perceptions of mass collectives affect political attitudes. Cambridge, England: Cambridge University Press.

[ref50] Ofcom (2023). News consumption in the UK. Available at: https://www.ofcom.org.uk/siteassets/resources/documents/research-and-data/tv-radio-and-on-demand-research/tv-research/news/news-consumption-2023/news-consumption-in-the-uk-2023 (Accessed November 25, 2024).

[ref51] Oreg S. Bayazit M. (2009). Prone to bias: development of a bias taxonomy from an individual differences perspective. Rev. Gen. Psychol. 13, 175–193. doi: 10.1037/a0015656

[ref52] Pachur T. Hertwig R. Steinmann F. (2012). How do people judge risks: availability heuristic, affect heuristic, or both? J. Exp. Psychol. Appl. 18, 314–330. doi: 10.1037/a0028279, PMID: 22564084

[ref53] Park S. Park J. Y. Kang J. H. Cha M. (2021). The presence of unexpected biases in online fact-checking. The Harvard Kennedy school misinformation. Review. doi: 10.37016/mr-2020-53

[ref54] Piro J. S. Anderson G. (2018). Intentional online discussions in teacher education. Teach. Educ. 53, 167–189. doi: 10.1080/08878730.2017.1419394

[ref55] Porter E. Wood T. J. (2021). The global effectiveness of fact-checking: evidence from simultaneous experiments in Argentina, Nigeria, South Africa, and the United Kingdom. Proc. Natl. Acad. Sci. 118:e2104235118. doi: 10.1073/pnas.2104235118, PMID: 34507996 PMC8449384

[ref56] PressGazette (2023). At 1,500 stories per day, Mail Online is UK’s most prolific news website. Available at: https://pressgazette.co.uk/media-audience-and-business-data/at-1500-stories-per-day-mail-online-is-uks-most-prolific-news-website/ (Accessed November 25, 2024).

[ref57] Rabbie J. M. Horwitz M. (1969). Arousal of ingroup-outgroup bias by a chance win or loss. J. Pers. Soc. Psychol. 13, 269–277. doi: 10.1037/h0028284, PMID: 5352845

[ref58] Rigotti E. Greco Morasso S. (2010). Comparing the argumentum model of topics to other contemporary approaches to argument schemes: the procedural and material components. Argumentation 24, 489–512. doi: 10.1007/s10503-010-9190-7

[ref59] Ross L. Greene D. House P. (1977). The “false consensus effect”: an egocentric bias in social perception and attribution processes. J. Exp. Soc. Psychol. 13, 279–301. doi: 10.1016/0022-1031(77)90049-X

[ref60] Ruffo G. Semeraro A. Giachanou A. Rosso P. (2023). Studying fake news spreading, polarisation dynamics, and manipulation by bots: a tale of networks and language. Comput. Sci. Rev. 47:100531. doi: 10.1016/j.cosrev.2022.100531

[ref61] Sádaba C. Salaverría R. Bringué X. (2023). Overcoming the age barrier: improving older adults’ detection of political disinformation with media literacy. Media Commun. 11, 113–123. doi: 10.17645/mac.v11i4.7090

[ref62] Saltor J. Barberia I. Rodríguez-Ferreiro J. (2023). Thinking disposition, thinking style, and susceptibility to causal illusion predict fake news discriminability. Appl. Cogn. Psychol. 37, 360–368. doi: 10.1002/acp.4008

[ref63] Şentürk Y. D. Ünver N. Demircan C. Egner T. Günseli E. (2024). The reactivation of task rules triggers the reactivation of task-relevant items. Cortex 171, 465–480. doi: 10.1016/j.cortex.2023.10.024, PMID: 38141571

[ref64] Soprano M. Roitero K. La Barbera D. Ceolin D. Spina D. Demartini G.. (2024). Cognitive biases in fact-checking and their countermeasures: a review. Inf. Process. Manag. 61:103672. doi: 10.1016/j.ipm.2024.103672

[ref65] Sperber D. Wilson D. (1986). Relevance: Communication and cognition (Vol. 142). Cambridge, MA: Harvard University Press.

[ref66] Statista (2024). Print Newspapers and Magazines – Worldwide. Available at: https://www.statista.com/outlook/amo/media/newspapers-magazines/print-newspapers-magazines/worldwide (Accessed November 25, 2024).

[ref67] Stencel M. Ryan E. Luther J. (2023). Misinformation spreads, but fact-checking has levelled off, Duke Reporters Lab. Available at: https://reporterslab.org/latest-news/ (Accessed November 25, 2024).

[ref68] Tandoc E. C. Lee J. Chew M. Tan F. X. Goh Z. H. (2021). Falling for fake news: the role of political bias and cognitive ability. Asian J. Commun. 31, 237–253. doi: 10.1080/01292986.2021.1941149

[ref69] Teovanović P. Lukić P. Zupan Z. Lazić A. Ninković M. Žeželj I. (2021). Irrational beliefs differentially predict adherence to guidelines and pseudoscientific practices during the COVID-19 pandemic. Appl. Cogn. Psychol. 35, 486–496. doi: 10.1002/acp.3770, PMID: 33362344 PMC7753549

[ref70] Tversky A. Kahneman D. (1973). Availability: a heuristic for judging frequency and probability. Cogn. Psychol. 5, 207–232. doi: 10.1016/0010-0285(73)90033-9, PMID: 38368678

[ref71] Tversky A. Kahneman D. (1974). Judgment under uncertainty: heuristics and biases: biases in judgments reveal some heuristics of thinking under uncertainty. Science 185, 1124–1131. doi: 10.1126/science.185.4157.1124, PMID: 17835457

[ref72] Ugille P. (2017). User generated content in the newsroom: professional and organisational constraints on participatory journalism. Westminster papers in communication and culture, 5(2).

[ref73] UK Paliament (2020), Breaking news? The future of UK Jounalism. Available at: https://publications.parliament.uk/pa/ld5801/ldselect/ldcomuni/176/17604.htm (Accessed November 25, 2024).

[ref74] Usearch (2024). How many news articles are published daily on the web?. Available at: https://usearch.com/blog/how-many-news-articles-are-published-daily-on-the-web (Accessed November 25, 2024).

[ref75] Van Schie E. C. Van Der Pligt J. (1995). Influencing risk preference in decision making: the effects of framing and salience. Organ. Behav. Hum. Decis. Process. 63, 264–275. doi: 10.1006/obhd.1995.1078

[ref76] Veldwijk J. Essers B. A. Lambooij M. S. Dirksen C. D. Smit H. A. De Wit G. A. (2016). Survival or mortality: does risk attribute framing influence decision-making behavior in a discrete choice experiment? Value Health 19, 202–209. doi: 10.1016/j.jval.2015.11.004, PMID: 27021754

[ref77] Waldman A. E. (2020). Cognitive biases, dark patterns, and the ‘privacy paradox’. Curr. Opin. Psychol. 31, 105–109. doi: 10.1016/j.copsyc.2019.08.025, PMID: 31590106

[ref78] Wallace L. E. Wegener D. T. Petty R. E. (2020). When sources honestly provide their biased opinion: Bias as a distinct source perception with independent effects on credibility and persuasion. Personal. Soc. Psychol. Bull. 46, 439–453. doi: 10.1177/0146167219858654, PMID: 31282841

[ref79] Walter N. Cohen J. Holbert R. L. Morag Y. (2020). Fact-checking: a meta-analysis of what works and for whom. Polit. Commun. 37, 350–375. doi: 10.1080/10584609.2019.1668894

[ref80] Walter N. Murphy S. T. (2018). How to unring the bell: a meta-analytic approach to correction of misinformation. Commun. Monogr. 85, 423–441. doi: 10.1080/03637751.2018.1467564

[ref81] Walter N. Tukachinsky R. (2020). A meta-analytic examination of the continued influence of misinformation in the face of correction: how powerful is it, why does it happen, and how to stop it? Commun. Res. 47, 155–177. doi: 10.1177/0093650219854600

[ref82] Wason P. (1960). On the failure to eliminate hypotheses in a conceptual task. Q. J. Exp. Psychol. 12, 129–140. doi: 10.1080/17470216008416717

[ref83] Wilson D. Sperber D. (2006). “Relevance theory” in The handbook of pragmatics. eds. Horn R. Ward G. (Blackwell, UK: Blackwell Publishing Ltd.) 606–632.

[ref84] Wu L. Morstatter F. Carley K. M. Liu H. (2019). Misinformation in social media: definition, manipulation, and detection. ACM SIGKDD Explorat. Newslett. 21, 80–90. doi: 10.1145/3373464.3373475

[ref85] Zarefsky D. (1997). “Definitions” in Argument in a time of change: Proceedings of the 10th biennial NCA/AFA summer conference on argumentation. ed. Klumpp J. F. (Annandale, VA: National Communication Association), 1–11.

[ref86] Zollo F. (2019). Dealing with digital misinformation: a polarised context of narratives and tribes. EFSA J. 17:e170720. doi: 10.2903/j.efsa.2019.e170720, PMID: 32626457 PMC7015504

[ref87] Zong Y. Guo X. (2022). An experimental study on anchoring effect of consumers’ price judgment based on consumers’ experiencing scenes. Front. Psychol. 13:794135. doi: 10.3389/fpsyg.2022.794135, PMID: 35211062 PMC8860899

