# Supplementary material for: Relevance theory for mapping cognitive biases in fact-checking: an argumentative approach
Source: Front Psychol. 2024 Dec 12;15:1468879. doi: 10.3389/fpsyg.2024.1468879 (PMC11670370; doi:10.3389/fpsyg.2024.1468879)
Supplement: Supplementary file 1 [file Table_1.DOCX]

Supplementary Material

# Supplementary Figures


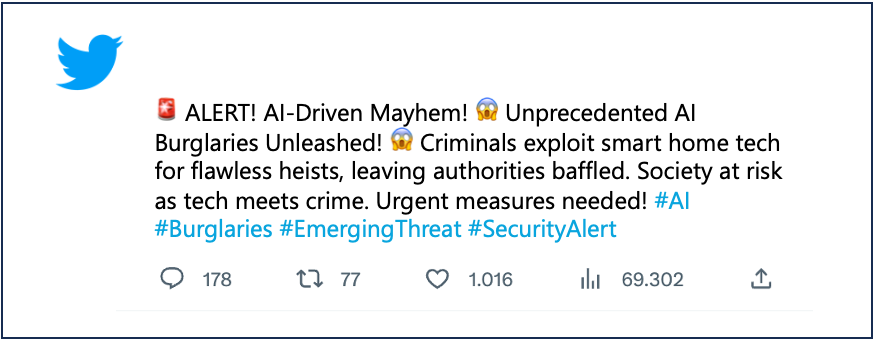


**Supplementary Figure 1.** News 1.


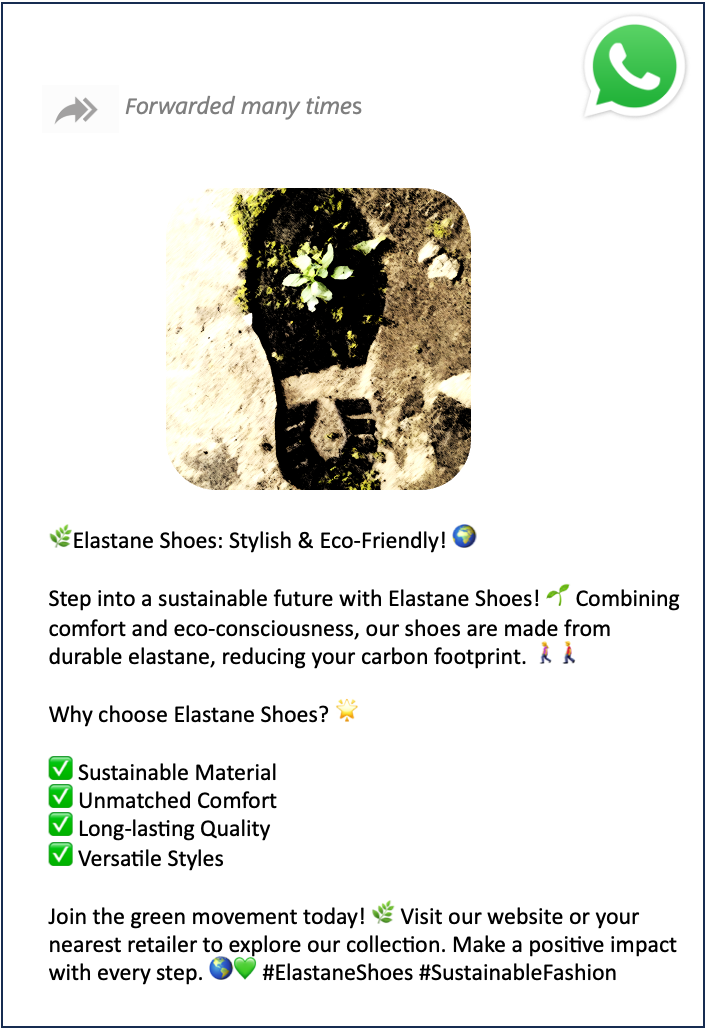


**Supplementary Figure 2.** News 2.


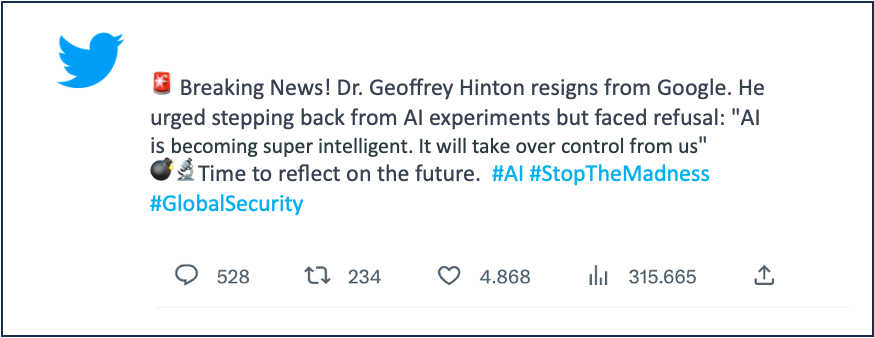


**Supplementary Figure 3.** News 3.

| 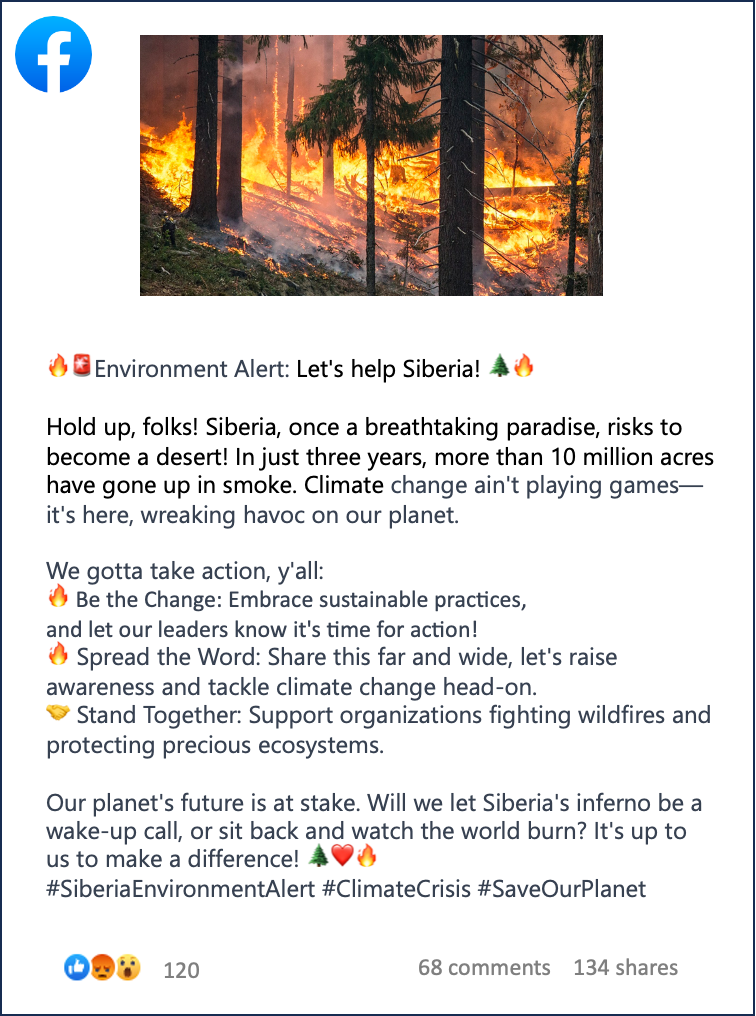  A) | 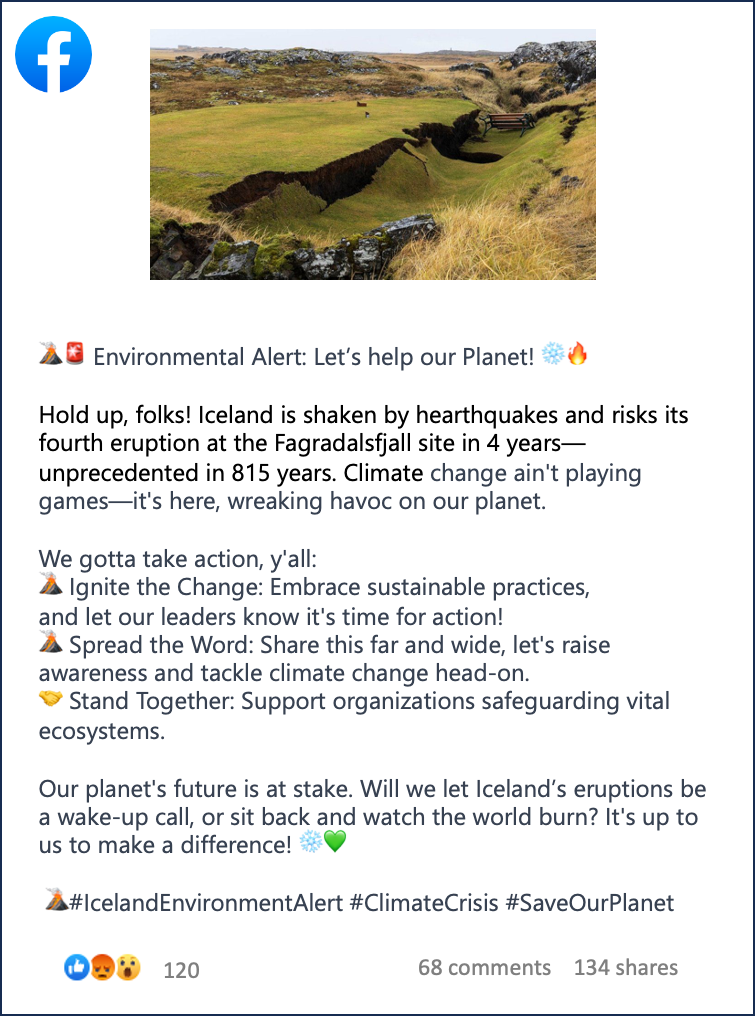  B) |
| --- | --- |

**Supplementary Figure 4.** News 4 presented during the focus group (A); News 4 presented during the interviews since the piece of News in Figure 4A was outdated (B).


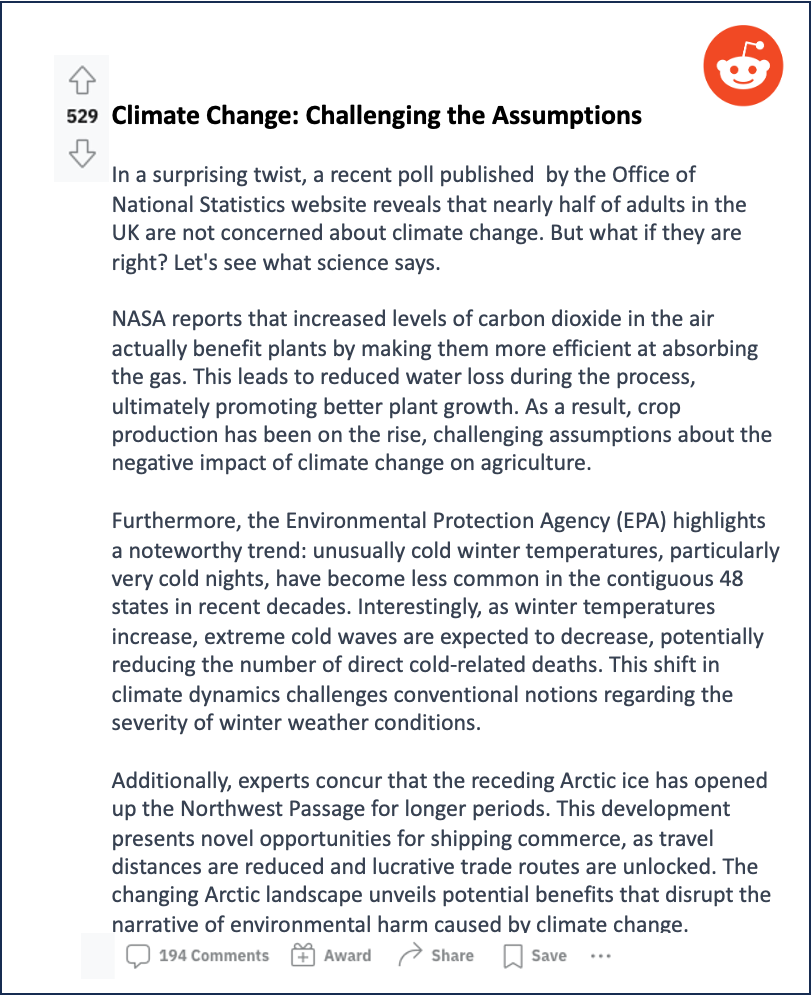


**Supplementary Figure 5.** News 5.


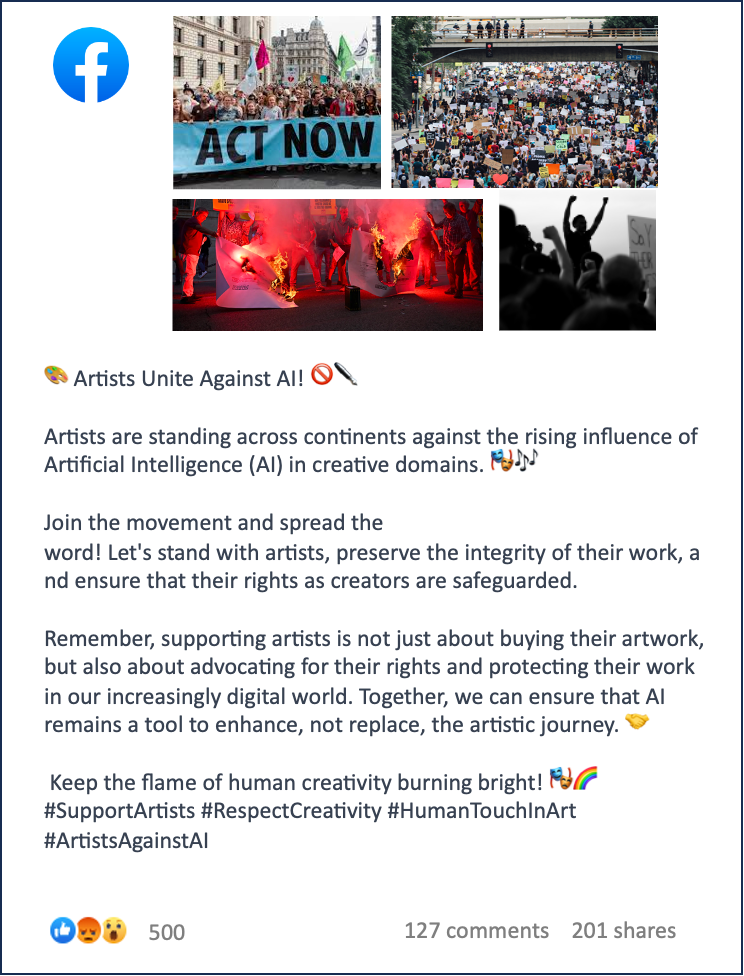


**Supplementary Figure 6.** News 6.


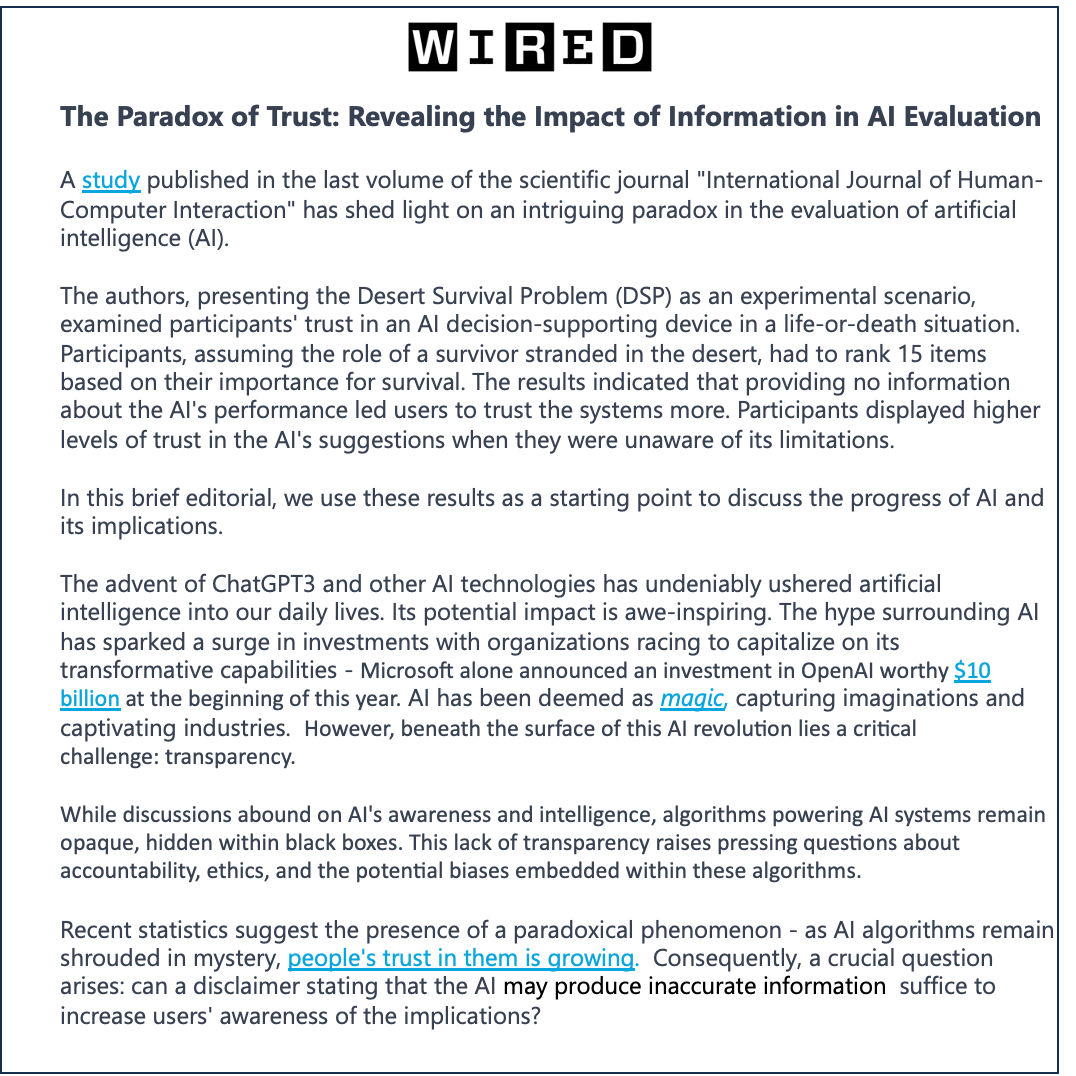


**Supplementary Figure 7.** News 7.


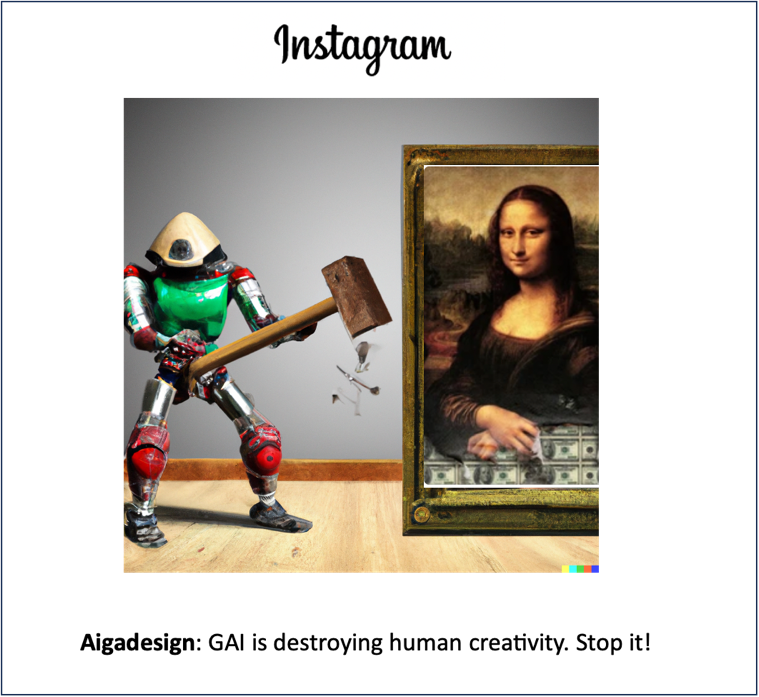


**Supplementary Figure 8.** News 8.
